# Umbilical Hemorrhage

**Published:** 1894-04

**Authors:** Thos. B. Grayson

**Affiliations:** Winkler, Texas


For Texas Medical Journal.

### UMBILICAL HEMORRHAGE.

THOS. B. GRAYSON, M. D., WINKLER, TEXAS.

ON February 23rd, I was called to the first case of umbilical hemorrhage I have ever seen in an active practice covering a period of about forty years. The child was a male, nine days old, weighed at birth between eight and nine pounds. The labor, so midwife reports, was easy and natural. The mother (colored) primapara, aged 18 years, stout and healthy, never in her life having had any sickness sufficiently severe to require the services of a physican. The cord in this case, fell off on the fifth day, and the midwife says every thing looked healthy and all right. Child had apparently done unusually well, nursed heartily, bowels and kidneys had acted freely and regularly, rested sweetly—till the morning of the eighth day, when a hemorrhagic oozing from the umbilicus was discovered, which continued to grow worse till I was called the succeeding morning. I found the child cold and pulseless (exhausted) and bleeding freely from umbilicus. The midwife had applied without favorable results cotton wadding saturated with liquor ferri sulphatis. I had no plaster of Paris at hand, so I prepared (which I think the better practice any way) through the umbilicus two needles at right angles and wound around each of them in the form of the figure 8 a waxed silk thread, and advised that they wrap it warmly, place hot bottles around, and give it frequently, in breast milk of a mixture of whiskey, turpentine and ergot, which I prepared. It bled no more after passing the needles. Before leaving I told them the child would die, which it did in about three hours. From the time the hemorrhage was discovered till death closed the scene was thirty-seven hours. The pathology in this condition is obscure. The prognosis is always grave. Five in every six, according to statistics, perish. It is useless to add that this child died from exhaustion.

---

**Why, of Course.**—STIVETS: The German investigators are experts in bacillus hunting, aren't they?"

WHIFFET: "Well, wouldn't you naturally expect a germ-man to cholera microbe?"

